
JOURNAL INFORMATION
==============================
NLM Title Abbreviation: ScientificWorldJournal
Journal ID: ScientificWorldJournal
NLM Journal ID: 101131163
Journal ID: TSWJ

The Scientific World Journal

ISSN: 2356-6140
EISSN: 1537-744X
Publisher: Wiley

ARTICLE INFORMATION
==============================
PMCID: PMC5720116
PMID: 21403976
DOI: 10.1100/tsw.2011.58
Article ID: 607423
Article version: 1
Subjects: Clinical Image

Teratoma of the Ovary with Sarcomatous Transformation and Pulmonary Metastases

Go Leonard H. T.
Corbridge Thomas C. *
Division of Pulmonary and Critical Care Medicine, Feinberg School of Medicine, Northwestern University, Chicago, IL, USA
*Thomas C. Corbridge: tcc734@northwestern.eduAcademic Editor: Daniel Batlle

Publication date: 2011
Electronic publication date: 2011 Mar 7
Volume: 11
First page: 584
Last page: 586
Received 2010 Nov 29; Revised 2011 Feb 3; Accepted 2011 Feb 4
Copyright: Copyright © 2011 Leonard H.T. Go and Thomas C. Corbridge.
Copyright year: 2011
License: This is an open access article distributed under the Creative Commons Attribution License, which permits unrestricted use, distribution, and reproduction in any medium, provided the original work is properly cited.
License URL: https://creativecommons.org/licenses/by/3.0/

Keywords: teratoma, germ-cell tumor, sarcoma

==============================
Clinical Image
*TheScientificWorldJOURNAL* (2011) 11, 584–586
ISSN 1537-744X; DOI 10.1100/tsw.2011.58

TheScientificWorldJOURNAL
www.thescientificworld.com

### Teratoma of the Ovary with Sarcomatous Transformation and Pulmonary Metastases

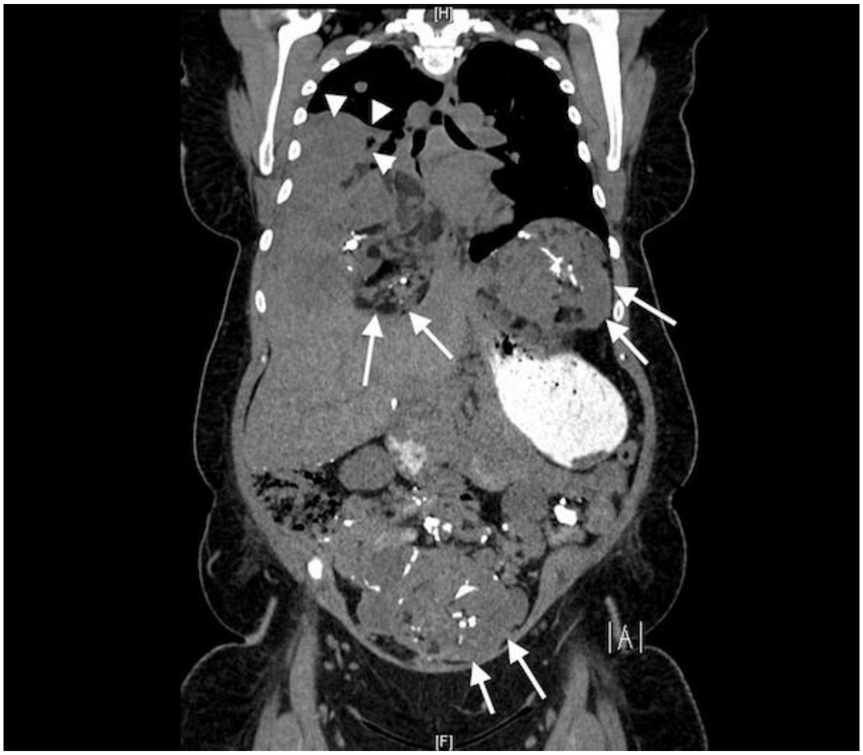

**FIGURE 1.** Computed tomogram of the chest and abdomen showing heterogeneous subdiaphragmatic and pelvic masses consistent with previously diagnosed teratoma (arrows) and a homogeneous mass contiguous with the teratoma in the right hemithorax, later found to be a sarcoma (arrowheads).

Leonard H.T. Go and Thomas C. Corbridge*
*Division of Pulmonary and Critical Care Medicine, Feinberg School of Medicine, Northwestern University, Chicago, IL*

**E-mail**: leonard-go@fsm.northwestern.edu, tcc734@northwestern.edu

*Received November 29, 2010; Revised February 3, 2011; Accepted February 4, 2011; Published March 7, 2011*

**KEYWORDS**: teratoma, germ-cell tumor, sarcoma

*Corresponding author.
©2011 with author.
Published by TheScientificWorld; www.thescientificworld.com

A 45-year-old woman with immature teratoma of the ovary previously treated with surgical resection and chemotherapy, with residual intra-abdominal disease shown to have mature elements only, underwent computed tomography of the chest and abdomen. The study demonstrated previously seen heterogeneous subdiaphragmatic and pelvic masses with calcifications (Fig. 1, arrows) consistent with teratoma, and revealed a new homogeneous mass contiguous with the teratoma on the right (arrowheads). In addition, numerous smaller pulmonary nodules were discovered (Fig. 2, arrows). The homogeneous mass was not seen on magnetic resonance imaging of the abdomen performed less than 10 months earlier (Fig. 3). The patient underwent biopsy of the newly discovered mass, with the finding of high-grade sarcoma. Teratomas are germ-cell tumors consisting of cells derived from all three germ-cell layers (ectoderm, mesoderm, and endoderm). Malignant transformation of one of the cell types within the teratoma can occur infrequently[1], as is believed to have occurred in the current case. Squamous cell carcinoma is the most frequently observed malignant cell type in this setting[2]. The occurrence of sarcomatous transformation is far less common, and may indicate a particularly poor prognosis[3]. Surgical resection may be considered for localized disease and chemotherapy determined by the malignant cell type observed can be given as well[4]. The described patient underwent treatment with doxorubicin, but was found later to have radiographic progression of disease. She died 6 months after the discovery of the sarcoma.

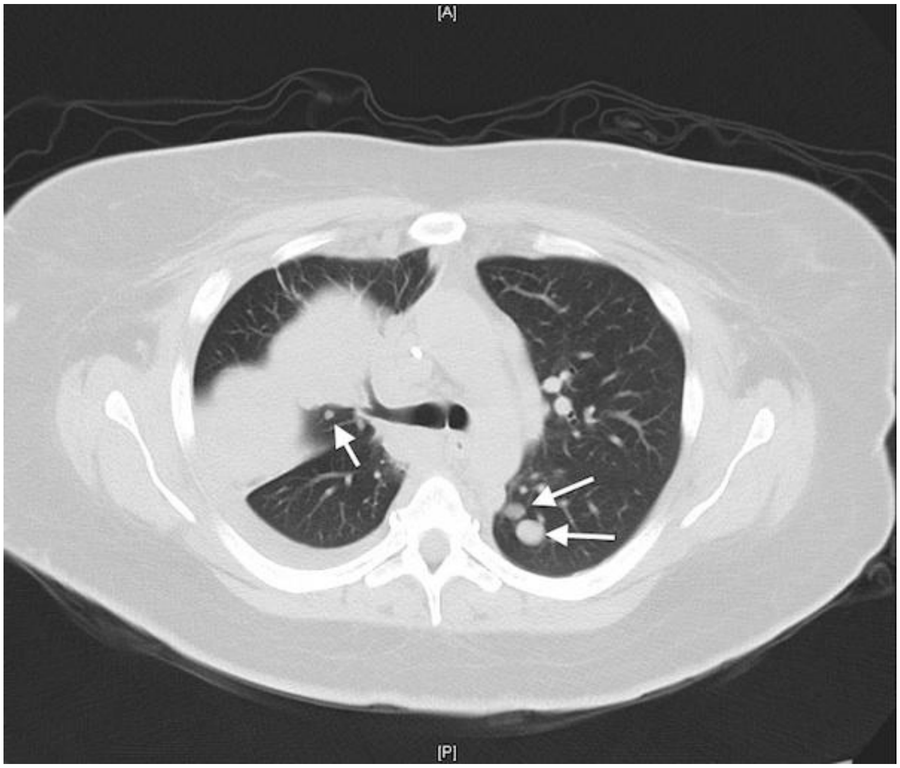

**FIGURE 2.** Computed tomogram of the chest demonstrating multiple pulmonary nodules thought to be metastases from the sarcoma (arrows).

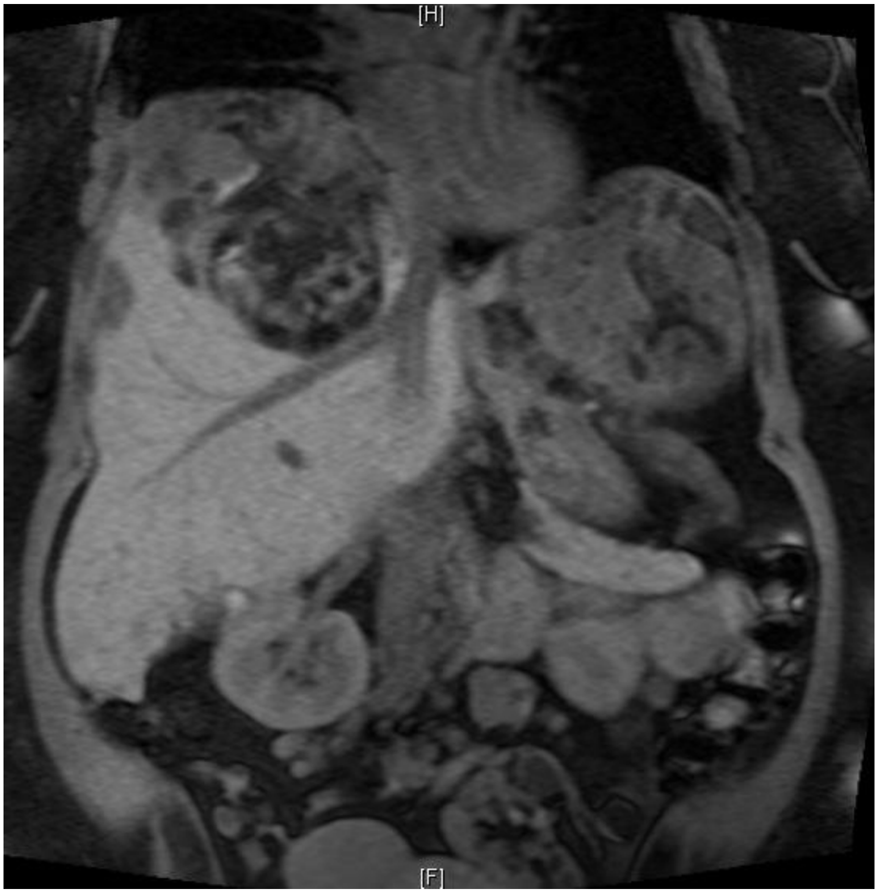

**FIGURE 3.** Magnetic resonance imaging of the abdomen performed 10 months prior to the study seen in Figs. 1 and 2. No homogeneous mass suggestive of the later-discovered sarcoma is seen.

### REFERENCES

1. Park, J.Y. et al. (2008) Malignant transformation of mature cystic teratoma of the ovary: experience at a single institution. *Eur. J. Obstet. Gynecol. Reprod. Biol.* **141(2),** 173–178.
2. Hackethal, A. et al. (2008) Squamous-cell carcinoma in mature cystic teratoma of the ovary: systematic review and analysis of published data. *Lancet Oncol.* **9(12)**, 1173–1180.
3. Malagon, H.D. et al. (2007) Germ cell tumors with sarcomatous components: a clinicopathologic and immunohistochemical study of 46 cases. *Am. J. Surg. Pathol.* **31(9),** 1356–1362.
4. El Mesbahi, O. et al. (2007) Chemotherapy in patients with teratoma with malignant transformation. *Eur. Urol.* **51(5),** 1306–1311; discussion 1311–1312.

**This article should be cited as follows:**

Go, L.H.T. and Corbridge, T.C. (2010) Teratoma of the ovary with sarcomatous transformation and pulmonary metastases. *TheScientificWorldJOURNAL* **11**, 584–586. DOI 10.1100/tsw.2011.58.
